# Supplementary material for: An Internet-Based Multi-Approach Intervention Targeting University Students Suffering from Psychological Problems: Design, Implementation, and Evaluation
Source: Int J Environ Res Public Health. 2022 Feb 25;19(5):2711. doi: 10.3390/ijerph19052711 (PMC8910035; doi:10.3390/ijerph19052711)
Supplement: Supplementary file 1 [file ijerph-19-02711-s001.zip › ijerph-1558276sup.pdf]

Supplementary File S1

2.5.1. Primary outcome measures

Beck Depression Inventory (BDI-II) [1,2].
The BDI-II is a self-report scale examining the current (past 2 weeks) severity of depression. It consists of 21 items, one per symptom, and participants are asked to choose one statement out of four to seven (coded 0, 1, 2, and 3). The depression severity is calculated by adding the value obtained in each of the 21 items. Values from 0 to 13 indicate minimal risk of depression; values from 14 to 19 indicate mild risk of depression; values from 20 to 28 indicate moderate risk of depression; values from 29 to 63 indicate severe risk of depression.

State-Trait Anxiety Inventory (STAI) [3,4].
The STAI is a 40-item self-report inventory divided into two sub-scales for both state- and trait-anxiety aimed at assessing and quantifying anxiety. The STAI is a Likert scale from 1 (not at all) to 4 (very much so) of 40 items. The first 20 items investigate current anxious symptoms, while the last 20 items refer to a more habitual and stable condition of the personality. The sum score, from 20 to 80, is obtained by inverting 19 item. Higher scores indicate higher level of anxiety, although a cut-off value of 39-40 has been suggested as clinically significant.

Italian Version of the Pittsburgh Sleep Quality Index (PSQI) [5,6].
The PSQI is a self-report 19-item questionnaire for the assessment of the overall sleep quality for the past month. It provides information about seven different components (subjective sleep quality, sleep latency, sleep duration, habitual sleep efficiency, sleep disturbances, use of sleep medications, daytime dysfunction) and a global score. Usually, a PSQI global score ≥ 5 is an indicator of clinically significant sleep pathological alteration in at least two components or of moderate difficulties in more than three components.

Brief version of the Columbia-Suicide Severity Rating Scale (C-SSRS) **modified** [7].
It consists of 4 items investigating current suicide risk and 5 items investigating life-time risk. The items are as follows: death wish ("Have you ever thought that you would be better off dead or wish you were dead?"); will of self-harm ("Have you ever wanted to harm yourself?"); suicidal ideation ("Have you ever thought about suicide?"); suicidal planning ("Have you ever thought about how you could take off your own life?"); attempted suicide ("Have you ever attempted suicide?").

School Burnout Inventory (SBI) [8,9].
The SBI is a 9-item scale investigating individual levels of burnout experience in students. The scale measures three dimensions: exhaustion in school, cynicism towards school, sense of inadequacy in school. All the items are rated on a 6-point Likert scale ranging from 1 (completely disagree) to 6 (strongly agree).

Difficulties in Emotion Regulation Scale (DERS-20) [10,11].
The DERS consists of 20 items investigating emotional regulation skills. Higher scores reflect greater impairment or dysregulation. No dysregulation: <90; moderate dysregulation: 91-105; high dysregulation: >105. Its six subscales are: (1) lack of emotional awareness (Awareness; "I am attentive to my feelings"); (2) lack of emotional clarity (Clarity; "I have difficulty making sense out of my feelings"); (3) difficulty regulating behavior when distressed (Impulse; "When I'm upset, I become out of control"); (4) difficulty engaging in goal-directed cognition and behavior when distressed (Goals; "When I'm upset, I have difficulty getting work done"); (5)

unwillingness to accept certain emotional responses (Non-acceptance; "When I'm upset, I become angry at myself for feeling that way"); and (6) lack of access to strategies for feeling better when distressed (Strategies; "When I'm upset, I believe there is nothing I can do to feel better").

Brief Interpersonal Reactivity Index (B-IRI) [12,13].
The B-IRI consists of 16 items, rated on a five-point Likert scale (from 0 = does not describe me at all, to 5 = it describes me completely), exploring four dimensions of empathy: 'Fantasy', referring to the tendency to imaginatively transpose oneself into the feelings and actions of fictitious characters; 'Perspective-Taking', evaluating the tendency to spontaneously adopt the psychological point of view of others; 'Empathic Concern', assessing the degree to which one experiences feelings of warmth, compassion and concern for an observed individual; and 'Personal Distress', evaluating the feelings of fear, apprehension and discomfort at witnessing the negative experiences of others. The scoring calculation will produce separate measurements for each factor, through a summation of the scores of the component items, and a total Index score corresponding to the sum of the subscale scores. Therefore, Fantasy: 3 + 7 + 11; Perspective Taking: 1 + 5 + 9 + 13 + 14; Empathic Concern: 4 + 8 + 12+ 16; Personal Distress: 2 + 6 + 10 + 15. The score for the items 4,8 and 12 should be reversed.

Brief Pain Inventory (BPI) [14,15].
The BPI gives two main scores: a pain severity score and a pain interference score. The pain severity score is calculated from the four items about pain intensity. Each item (rated from 0, no pain, to 10, pain as bad as you can imagine) contributes with the same weight to the final score, ranging from 0 to 40. Cut-off values for mild, moderate, and severe pain: 0 = no pain; 1–3 = mild pain; 4–6 = moderate pain; ≥7 = severe pain. The pain interference score corresponds to the items on pain interference. The seven sub-items (rated from 0, does not interfere, to 10, completely interferes) contribute with the same weight to the final score, ranging from 0 to 70.

World Health Organization Quality of Life - short version (WHOQOL-BREF) [16].
The WHOQOL-BREF is a 26-item instrument consisting of four domains: physical health (7 items), psychological health (6 items), social relationships (3 items), and environmental health (8 items); it also contains QOL and general health items. Each individual item is scored from 1 to 5 on a five-point ordinal scale. The scores are then transformed linearly to a 0–100-scale. A cut-off < 60 indicates a worse quality of life. The physical health domain includes items on mobility, daily activities, functional capacity, energy, pain, and sleep. The psychological domain includes self-image, negative thoughts, positive attitudes, self-esteem, mentality, learning ability, memory concentration, religion, and the mental status. The social relationships domain contains questions on personal relationships, social support, and sex life. The environmental health domain contains issues related to financial resources, safety, health and social services, living physical environment, opportunities to acquire new skills and knowledge, recreation, general environment (noise, air pollution, etc.), and transportation.

2.5.2. Secondary outcome measures

Visual Analogue Scales (VAS) [17].
The VAS evaluates on a 0-10 scale the intensity of somatic and psychological pain (current, usual and maximal perceived in the last 15 days). A score > 2 indicates high current physical pain and a score > 3 and > 4 indicates high habitual physical pain and

maximum physical pain in the last 15 days, respectively. For high levels of current mental pain, a cut off value > 7 will be considered, while for habitual and maximum mental pain > 7 and > 8 respectively.

Insomnia Severity Index (ISI) [18].
The ISI is a seven-item questionnaire widely used to quantify insomnia by evaluating some sleep aspects (i.e., sleep onset, sleep maintenance, early morning awakenings, sleep dissatisfaction, interference of sleep alterations with daytime functioning, sleep problems reported by others, level of distress secondary by the sleep alterations), referred to the past two weeks. The ISI total score ranges between 0 and 28, with a higher score indicating greater insomnia severity. More specifically, scores between 0 and 7 indicate non-significant insomnia, scores between 8 and 14 indicate subthreshold insomnia, scores between 15 and 21 indicate insomnia of moderate severity, and scores higher than 22 indicate severe insomnia.

Toronto Alexithymia Scale (TAS-20) [19,20].
The TAS-20 is a self-report 20-item scale. Items are rated using a 5-point Likert scale (1 = strongly disagree and 5 = strongly agree). There are 5 items that are negatively keyed (items 4, 5, 10, 18 and 19). The total alexithymia score is the sum of responses to all 20 items. The cutoff scoring: equal to or less than 51 = non-alexithymia, equal to or greater than 61 = alexithymia. Scores of 52 to 60 = possible alexithymia.

Coping Orientation to the Problems Experienced (COPE-NVI-25) [21,22].
The COPE-NVI-25 consists of 25 items evaluating how often the subject uses that particular coping process in difficult or stressful situations (the individuals should think about how they usually behave in stressful situations). Response choices varied from 1 (I usually don't do this at all) to 4 (I usually do this a lot). Five independent dimensions are present: social support ("I try to get advice from someone about what to do"), avoidance strategies ("I refuse to believe that it has happened"), positive attitude ("I look for something good in what is happening"), problem solving ("I focus on dealing with this problem, and if necessary let other things slide a little") and turning to religion ("I pray more than usual").

Life Events Checklist (LEC) [23].
LEC is a self-report measure designed to screen for potentially traumatic events in a respondent's lifetime. The LEC assesses exposure to 16 stressful events. Respondents indicate varying levels of exposure to each type of potentially traumatic event included on a 6-point nominal scale, and may endorse multiple levels of exposure to the same trauma type. The LEC does not yield a total score or composite score.

Reasons For Living Inventory (RFLI) [24].
This scale consists of 48 items investigating the subject's reasons for living. It has six valid and reliable subscales: Survival and Coping Beliefs (SCB), Responsibility to Family (RF), Child Related Concerns (CRC), Fear of Suicide (FS), Fear of Social Disapproval (FSD), and Moral Objections (MO). The 48 items are scored on a 6-point scale ranging from (1) "not at all important" to (6) "extremely important". Higher scores represent more reasons to live.

COVID-19-related questions compiled according to the International Association for Suicide Prevention (IASP) guidelines for suicide prevention research [25].

1. Beck, A.T.; Steer, R.A.; Brown, G.K. *Beck Depression Inventory (BDI-II)*; Pearson: London, UK, **1996**; Volume 10.

2. Ghisi, M.; Flebus, G.B.; Montano, A.; Sanavio, E.; Sica, C. Manuale BDI-II. *Beck Depression Inventory–II. Organizzazioni Speciali, Firenze* **2006**.
3. Pedrabissi, L.; Santinello, M. Verifica Della Validità Dello STAI Forma Y Di Spielberger. *Giunti Organizzazioni Speciali* **1989**, *191–192*, 11–14.
4. Spielberger, C.D. *State-Trait Anxiety Inventory for Adults*; Consulting Psychologists Press: Palo Alto, CA, USA, **1983**.
5. Buysse, D.J.; Reynolds III, C.F.; Monk, T.H.; Berman, S.R.; Kupfer, D.J. The Pittsburgh Sleep Quality Index: A New Instrument for Psychiatric Practice and Research. *Psychiatry research* **1989**, *28*, 193–213.
6. Curcio, G.; Tempesta, D.; Scarlata, S.; Marzano, C.; Moroni, F.; Rossini, P.M.; Ferrara, M.; De Gennaro, L. Validity of the Italian Version of the Pittsburgh Sleep Quality Index (PSQI). *Neurological Sciences* **2013**, *34*, 511–519.
7. Posner, K.; Brown, G.K.; Stanley, B.; Brent, D.A.; Yershova, K.V.; Oquendo, M.A.; Currier, G.W.; Melvin, G.A.; Greenhill, L.; Shen, S. The Columbia–Suicide Severity Rating Scale: Initial Validity and Internal Consistency Findings from Three Multisite Studies with Adolescents and Adults. *American journal of psychiatry* **2011**, *168*, 1266–1277.
8. Fiorilli, C.; Galimberti, V.; De Stasio, S.; Di Chiacchio, C.; Albanese, O. L'utilizzazione Dello School Burnout Inventory (SBI) Con Studenti Italiani Di Scuola Superiore Di Primo e Secondo Grado. *Psicologia clinica dello sviluppo* **2014**, *18*, 403–424.
9. Salmela-Aro, K.; Kiuru, N.; Leskinen, E.; Nurmi, J.-E. School Burnout Inventory (SBI) Reliability and Validity. *European journal of psychological assessment* **2009**, *25*, 48–57.
10. Gratz, K.L.; Roemer, L. Multidimensional Assessment of Emotion Regulation and Dysregulation: Development, Factor Structure, and Initial Validation of the Difficulties in Emotion Regulation Scale. *Journal of psychopathology and behavioral assessment* **2004**, *26*, 41–54.
11. Lausi, G.; Quaglieri, A.; Burrai, J.; Mari, E.; Giannini, A.M. Development of the DERS-20 among the Italian Population: A Study for a Short Form of the Difficulties in Emotion Regulation Scale. *Mediterranean Journal of Clinical Psychology* **2020**, *8*, https://doi.org/10.6092/2282-1619/mjcp-2511.
12. Davis, M.H. A Multidimensional Approach to Individual Differences in Empathy. The University of Texas: Austin, TX, USA, **1980**.
13. Ingoglia, S.; Lo Coco, A.; Albiero, P. Development of a Brief Form of the Interpersonal Reactivity Index (B–IRI). *Journal of Personality Assessment* **2016**, *98*, 461–471.
14. Caraceni, A.; Mendoza, T.R.; Mencaglia, E.; Baratella, C.; Edwards, K.; Forjaz, M.J.; Martini, C.; Serlin, R.C.; de Conno, F.; Cleeland, C.S. A Validation Study of an Italian Version of the Brief Pain Inventory (Breve Questionario per La Valutazione Del Dolore). *Pain* **1996**, *65*, 87–92, doi:10.1016/0304-3959(95)00156-5.
15. Cleeland, C.S.; Ryan, K. *The Brief Pain Inventory*; The University of Texas MD Anderson Cancer Center, Pain Research Group: Houston, TX, USA, **1991**; 143–147.
16. WHOQOL Group. Development of the World Health Organization WHOQOL-BREF Quality of Life Assessment. *Psychol. Med.* **1998**, *28*, 551–558.
17. Olié, E.; Guillaume, S.; Jaussent, I.; Courtet, P.; Jollant, F. Higher Psychological Pain during a Major Depressive Episode May Be a Factor of Vulnerability to Suicidal Ideation and Act. *Journal of Affective Disorders* **2010**, *120*, 226–230.
18. Castronovo, V.; Galbiati, A.; Marelli, S.; Brombin, C.; Cugnata, F.; Giarolli, L.; Anelli, M.M.; Rinaldi, F.; Ferini-Strambi, L. Validation Study of the Italian Version of the Insomnia Severity Index (ISI). *Neurological Sciences* **2016**, *37*, 1517–1524.
19. Bagby, R.M.; Parker, J.D.; Taylor, G.J. The Twenty-Item Toronto Alexithymia Scale—I. Item Selection and Cross-Validation of the Factor Structure. *Journal of psychosomatic research* **1994**, *38*, 23–32.
20. Bressi, C.; Taylor, G.; Parker, J.; Bressi, S.; Brambilla, V.; Aguglia, E.; Allegranti, I.; Bongiorno, A.; Giberti, F.; Bucca, M. Cross Validation of the Factor Structure of the 20-Item Toronto Alexithymia Scale: An Italian Multicenter Study. *Journal of psychosomatic research* **1996**, *41*, 551–559.
21. Carver, C.S.; Scheier, M.F.; Weintraub, J.K. Assessing Coping Strategies: A Theoretically Based Approach. *Journal of personality and social psychology* **1989**, *56*, 267.

22. Foà, C.; Tonarelli, A.; Caricati, L.; Fruggeri, L. COPE-NVI-25: Validazione Italiana Della Versione Ridotta Della Coping Orientation to the Problems Experienced (COPE-NVI). *Psicol. Salut.* **2015**, 123–140. https://doi.org/10.3280/PDS2015-002007.
23. Gray, M.J.; Litz, B.T.; Hsu, J.L.; Lombardo, T.W. Psychometric Properties of the Life Events Checklist. *Assessment* **2004**, *11*, 330–341.
24. Linehan, M.M.; Goodstein, J.L.; Nielsen, S.L.; Chiles, J.A. Reasons for Staying Alive When You Are Thinking of Killing Yourself: The Reasons for Living Inventory. *Journal of consulting and clinical psychology* **1983**, *51*, 276.
25. International Association for Suicide Prevention (ICSPRC). Advice on Questions/Survey Tools Relevant to Suicide Prevention Research in Relation to COVID-19. Available online: https://iasp.info/wp-content/uploads/ICSPRC_COVID_advice_on_questions.pdf (accessed on 05.03.2021).
