# Supplementary material for: An Internet-Based Multi-Approach Intervention Targeting University Students Suffering from Psychological Problems: Design, Implementation, and Evaluation
Source: Int J Environ Res Public Health. 2022 Feb 25;19(5):2711. doi: 10.3390/ijerph19052711 (PMC8910035; doi:10.3390/ijerph19052711)
Supplement: Supplementary file 1 [file ijerph-19-02711-s001.zip › ijerph-1558276sup2.pdf]

	Control Condition (N = 100)		MindBlooming Intervention (N = 100)
**T0**	– Written introduction<br><br>– Global screening<br>– Screening results: assignment of two areas of distress XY<br>X = area of distress 1<br>Y = area of distress 2<br><br>*After three days (max):*	**T0**	– Written and video introduction<br><br>– Global screening<br>– Screening results: assignment of two modules XY<br>X = module 1<br>Y = module 2<br><br>*After three days (max):*
**WEEK 1**	Educational material and resources on Qualtrics<br><br>– Weekly screening about two areas of distress XY	**WEEK 1**	Psychoeducation XY<br>Exercises WEEK1 X<br>Exercises WEEK1 Y<br><br>• Day 1 – Daily screening<br>• Day 2 – Daily screening<br>• Day 3 – Daily screening<br>• Day 4 – Daily screening<br>• Day 5 – Daily screening<br>• Day 6 – Daily screening<br>• Day 7 – Daily screening<br>– Weekly screening about two modules XY
**WEEK 2**	– Weekly screening about two areas of distress XY	**WEEK 2**	Psychoeducation XY<br>Exercises WEEK2 X<br>Exercises WEEK2 Y<br><br>• Day 8 – Daily screening<br>• Day 9 – Daily screening<br>• Day 10 – Daily screening<br>• Day 11 – Daily screening<br>• Day 12 – Daily screening<br>• Day 13 – Daily screening<br>• Day 14 – Daily screening<br>– Weekly screening about two modules XY
**WEEK 3**	– Weekly screening about two areas of distress XY	**WEEK 3**	Psychoeducation XY<br>Exercises WEEK3 X<br>Exercises WEEK3 Y<br><br>• Day 15 – Daily screening<br>• Day 16 – Daily screening<br>• Day 17 – Daily screening<br>• Day 18 – Daily screening<br>• Day 19 – Daily screening<br>• Day 20 – Daily screening<br>• Day 21 – Daily screening<br>– Weekly screening about two modules XY
**WEEK 4**	– Weekly screening about two areas of distress XY	**WEEK 4**	Psychoeducation XY<br>Exercises WEEK4 X<br>Exercises WEEK4 Y<br><br>• Day 22 – Daily screening<br>• Day 23 – Daily screening<br>• Day 24 – Daily screening<br>• Day 25 – Daily screening<br>• Day 26 – Daily screening<br>• Day 27 – Daily screening<br>• Day 28 – Daily screening<br>– Weekly screening about two modules XY

	Control Condition (N = 100)			MindBlooming Intervention (N = 100)
WEEK 5	– Weekly screening about two areas of distress XY		WEEK 5	Psychoeducation XY<br>Exercises WEEK5 X<br>Exercises WEEK5 Y<br>• Day 29 – Daily screening<br>• Day 30 – Daily screening<br>• Day 31 – Daily screening<br>• Day 32 – Daily screening<br>• Day 33 – Daily screening<br>• Day 34 – Daily screening<br>• Day 35 – Daily screening<br>– Weekly screening about two modules XY
WEEK 6	– Weekly screening about two areas of distress XY and relational issues		WEEK 6	Psychoeducation about the relational problem module (for everyone)<br>Exercises WEEK6<br>• Day 36 – Daily screening<br>• Day 37 – Daily screening<br>• Day 38 – Daily screening<br>• Day 39 – Daily screening<br>• Day 40 – Daily screening<br>• Day 41 – Daily screening<br>• Day 42 – Daily screening<br>– Weekly screening about two modules XY and relational problems
WEEK 7	– Weekly screening about two areas of distress XY		WEEK 7	Psychoeducation XY<br>Exercices WEEK7 XY<br>(recap and maintenance)<br>• Day 43 – Daily screening<br>• Day 44 – Daily screening<br>• Day 45 – Daily screening<br>• Day 46 – Daily screening<br>• Day 47 – Daily screening<br>• Day 48 – Daily screening<br>• Day 49 – Daily screening<br>– Weekly screening about two modules XY
T1	– Global screening*<br>+ CSQ-8 and SUS	POST TREATMENT	T1	– Global screening*<br>+ CSQ-8 and SUS
T2	– Global screening*	12 WEEKS AFTER	T2	– Global screening*
T3	– Global screening*	24 WEEKS AFTER	T3	– Global screening*

Global screening* = except inclusion/exclusion criteria and socio-demographic questions
